# Flying under the radar – impact and factors influencing asymptomatic DENV infections

**DOI:** 10.3389/fcimb.2023.1284651

**Published:** 2023-11-24

**Authors:** Paulo Henriques, Alexandra Rosa, Helena Caldeira-Araújo, Pedro Soares, Ana Margarida Vigário

**Affiliations:** ^1^ Projecto Medicina, Faculdade de Ciências da Vida, Universidade da Madeira, Funchal, Portugal; ^2^ CQM-Centro de Química da Madeira, Universidade da Madeira, Funchal, Portugal; ^3^ Department of Biology, CBMA (Centre of Molecular and Environmental Biology), Braga, Portugal; ^4^ Department of Biology, Institute of Science and Innovation for Bio-Sustainability (IB-S), University of Minho, Braga, Portugal; ^5^ Instituto de Medicina Molecular João Lobo Antunes, Faculdade de Medicina, Universidade de Lisboa, Lisboa, Portugal

**Keywords:** dengue, DENV infection, asymptomatic infections, host factors, flavivirus infections

## Abstract

The clinical outcome of DENV and other Flaviviruses infections represents a spectrum of severity that ranges from mild manifestations to severe disease, which can ultimately lead to death. Nonetheless, most of these infections result in an asymptomatic outcome that may play an important role in the persistent circulation of these viruses. Also, although little is known about the mechanisms that lead to these asymptomatic infections, they are likely the result of a complex interplay between viral and host factors. Specific characteristics of the infecting viral strain, such as its replicating efficiency, coupled with host factors, like gene expression of key molecules involved in the immune response or in the protection against disease, are among crucial factors to study. This review revisits recent data on factors that may contribute to the asymptomatic outcome of the world’s widespread DENV, highlighting the importance of silent infections in the transmission of this pathogen and the immune status of the host.

## 1 Introduction

In the last few decades, flaviviral diseases have become progressively more common in human populations, propelled by spread into previously absent regions, representing nearly 30% of all emerging infectious diseases in humans in the early 2000s (Jones et al., 2008). Examples include the introduction of dengue virus serotype 1 (DENV1) in Madeira (Portugal) in 2012, resulting in an outbreak with 2,168 probable cases (European Centre Disease Control, 2014), and Zika virus (ZIKV) in South America in 2015 (Campos et al., 2015), causing an outbreak of 1.3 million suspected cases across at least 33 territories (Hennessey et al., 2016; World Health Organization, 2016). The re-emergence of flaviviral diseases in regions where they had previously been controlled or eradicated, namely DENV in Brazil in 1981 (Osanai et al., 1983) and yellow fever virus (YFV) in Kenia in 1992 (Okello et al., 1993), also contributed to a global increase of cases.

Clinical manifestations of flaviviral infections range from mild to severe disease (Hollidge et al., 2010). Nevertheless, it is likely that up to 95% of these infections in humans are asymptomatic (Balmaseda et al., 2010; Johansson et al., 2014). Asymptomatic infections are highly significant in epidemiology since their estimate is crucial for determining the real burden of infection and the risk of transmission in a population. Furthermore, little is known about the underlying factors of asymptomatic outcome across flaviviral infections. Here, we aim to review recent data on factors contributing to the asymptomatic outcome of the widespread flaviviral DENV infection, and the importance of silent infections in transmission and the immune status of the host.

## 2 Classification and transmission of Flaviviruses

**Figure 1:**
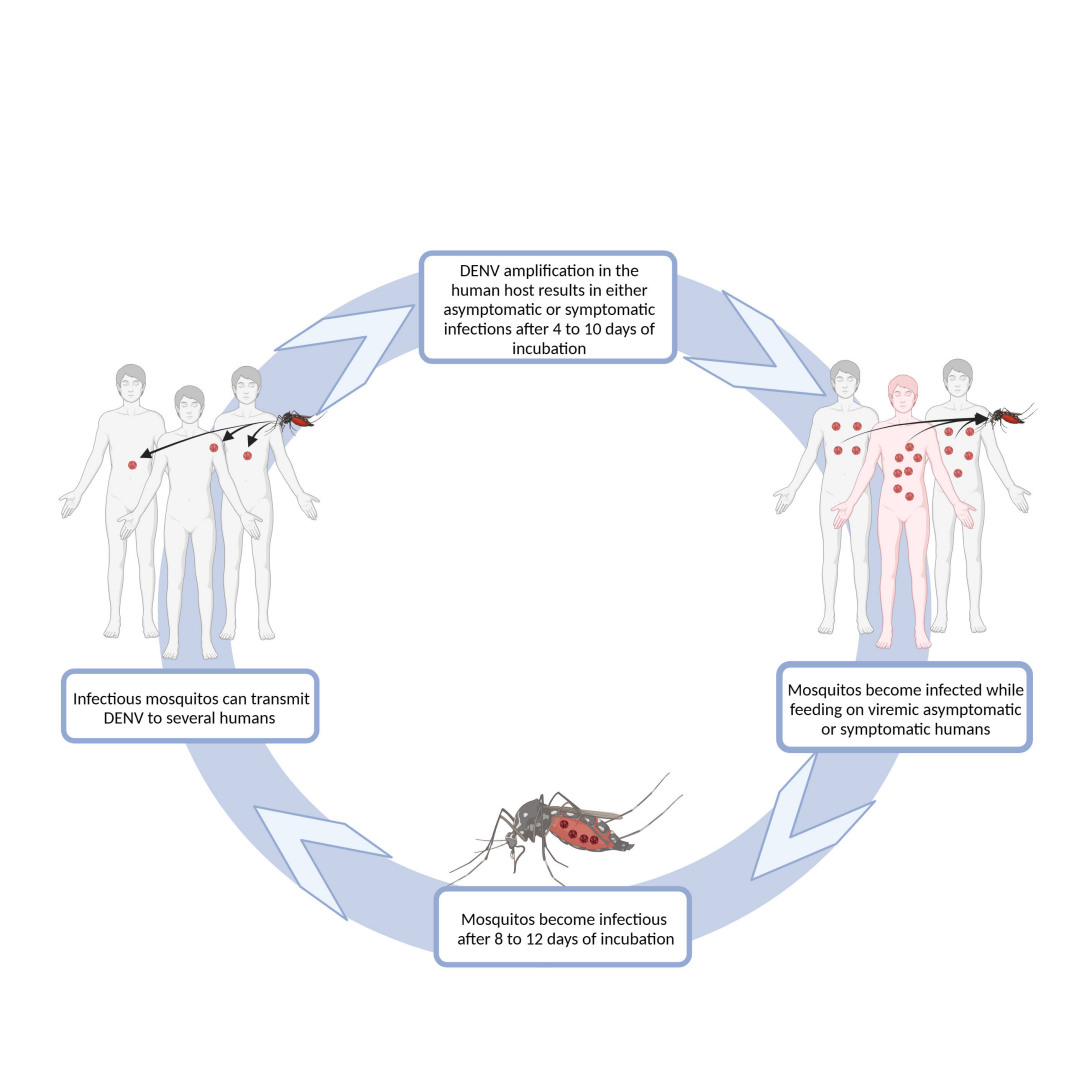
Schematic representation of DENV transmission. DENV infection in humans is initiated after the virus is delivered into the host’s skin, during a blood meal of an infected female *Aedes* sp. mosquito. One mosquito can infect several humans, as it can bite several times before completing oogenesis. Some of the infected humans will develop dengue symptoms after 4-10 days of virus incubation, while others will remain asymptomatic or have an inapparent infection (World Health Organization, 2009). Both symptomatic and asymptomatic humans may have sufficiently high viremia to infect mosquitoes during their feeding. After a period of 8-12 days, mosquitoes are able to infect humans (World Health Organization, 2009). Diagram was made using BioRender.com.

Flavivirus is a genus of viruses of the Flaviviridae family, which share common features such as the presence of an envelope, an icosahedral nucleocapsid, and a +ssRNA genome. This genus includes the DENV, YFV, ZIKV, West Nile Virus (WNV), Japanese Encephalitis Virus (JEV), as well as several other viruses causing encephalitis (Shi, 2012). Flaviviruses are transmitted from infected hematophagous arthropods to different enzootic vertebrate reservoir hosts, such as domestic animals and humans, henceforth classified as arboviruses. When viremia is sufficiently high in these amplifying hosts, permissive arthropods become infected during a blood meal, transmitting it further on to a new host in a subsequent meal, allowing the maintenance of the cycle. For WNV (Bowen and Nemeth, 2007) and JEV (Weaver and Barrett, 2004), humans are often dead-end hosts, given that their typical low viremia unlikely allows reinfections by feeding arthropods and the chances of reencountering a vector in areas of low population density are scarce, therefore interrupting the viral lifecycle (Hollidge et al., 2010). On the contrary, DENV, YFV, and ZIKV can establish active transmission cycles in areas with high population density, using humans as the primary amplifying host (Nimmannitya et al., 1969; Padbidri and Gnaneswar, 1979; Weaver and Barrett, 2004; Barrett and Higgs, 2007; Saiz et al., 2016), becoming highly relevant as human pathogens (**Figure 1**).

Even though DENV is completely adapted to urban cycles, there is ample evidence that DENV infects non-human primates (Gwee et al., 2021), thought to act as amplification hosts for enzootic transmission (Vasilakis et al., 2011; Valentine et al., 2019). Anti-DENV antibodies have been largely detected in non-human primates, including those living in urban and peri-urban areas, namely zoos (Valentine et al., 2019; Gwee et al., 2021). However, the range of human infections caused by sylvatic DENV strains are still quite unknown (reviewed in Vasilakis et al., 2011). Nonetheless, sylvatic cycles can potentially act as a reservoir for the virus, contributing to DENV recurrence after an epidemic and following the decline of human population herd immunity, or even to the development of new strains with increased (or decreased) virulence for humans (Valentine et al., 2019).

## 3 The DENV journey in the mammalian host

Following the bite of an infected arthropod, it is likely that the inoculation of DENV into the dermis and epidermis of the mammalian host results in infection (Guzman et al., 2016) since, besides skin dendritic cells (DCs), other phagocytic cells such as monocytes and macrophages, as well as keratinocytes, are all viable targets for the virus (Wu et al., 2000; Marovitch et al., 2001; Surasombatpattana et al., 2011). Infected cells can then migrate from the initial site of infection to lymph nodes, where the adaptive immune response is initiated. The resulting activation of effector T cells (Silberberg-Sinakin et al., 1976; Macatonia et al., 1987) prompts the recruitment of other monocytes and macrophages, that become further targets for the virus (Guzman et al., 2016) (see **Box 1** for details on immune response against DENV). The dissemination of the infection throughout the lymphatic system is then facilitated by the subsequent infection of other cells of the mononuclear lineage, including monocytes, myeloid DCs, and liver and splenic macrophages (Marovitch et al., 2001).

Once inside the mammalian host, all flaviviruses follow similar steps in their replication cycle [reviewed in Fernandez-Garcia et al. (2009)]. The DENV replication cycle initiates with the binding of the envelope (E) proteins of mature viral particles to the host target cell receptors, followed by internalization through endocytosis. *In vitro*, DENV showed the capacity to use a wide variety of cellular receptors to attach and enter target cells, although the relevance to natural human infection is not fully established [reviewed in Cruz-Oliveira et al. (2015)]. For example, *in vitro* DENV infection has been positively correlated with an increased expression of dendritic-cell-specific intercellular adhesion molecule 3’-grabbing non-integrin (DC-SIGN, CD209) in human monocytes-derived DCs and of its homologue L-SIGN (CD209L) in the endothelial cells of the liver and lymph nodes (Navarro-Sanchez et al., 2003; Tassaneetrithep et al., 2003). The lipopolysaccharide-binding protein (LPS) receptor (CD14) and the macrophage mannose receptor (CD206) were shown to be involved in DENV binding to monocytes/macrophages (Chen et al., 1999; Miller et al., 2008). Other candidate receptors such as the heparan sulphate receptor, expressed in hepatocytes, and the heat-shock proteins HSP70 and HSP90 have also been described as targeted by DENV *in vitro* (Germi et al., 2002; Reyes-Del Valle et al., 2005). Also, apolipoprotein-A-I (ApoA-I) scavenger receptor class b type I (SR-BI) seems to facilitate DENV entry into cells (Li et al., 2013), and endocytosis via LDL receptor has been reported in several flaviviruses (Agnello et al., 1999). After the humoral response has been established, the virus may also form a complex with non-neutralizing antibodies, facilitating its entrance into cells expressing receptors for the Fc region of the antibodies, like monocytes. This phenomenon is called antibody-dependent enhancement (ADE) and it is likely implicated in the pathogenesis, especially during secondary heterologous DENV infections [reviewed in Halstead (2003)].

Once inside the host cell, viral genomic (+)ssRNA is translated into a long polyprotein, which is cleaved into individual E, membrane (M), and capsid (C) structural proteins, and several non-structural (NS) proteins. Viral RNA, together with C proteins, will assemble into new nucleocapsids. The precursor forms of the membrane (prM) and E proteins are embedded into the endoplasmic reticulum membrane and will surround the newly formed nucleocapsids, constituting immature viral particles. The maturation of DENV particles will occur along the secretory pathway and, eventually, the infective mature DENV exits the host cells via exocytosis [reviewed in Guzman et al. (2016)].

## 4 The outcomes of DENV infections

The clinical outcome of DENV infections, for which 4 circulating serotypes are known (DENV1-4), results from the interplay between host and pathogen factors. As for other flaviviral infections, the clinical manifestations may range from a mild influenza-like illness to severe disease, possibly resulting in long-term physical impairment or even death (Hollidge et al., 2010).

According to WHO guidelines until 2009, symptomatic dengue was classified as dengue fever (DF), dengue hemorrhagic fever (DHF), and dengue shock syndrome (DSS), the latter being the most severe form. Following 2009, WHO classification system divides DENV infection in non-severe dengue (with or without warning signs) and severe dengue, depending on the severity of the clinical manifestations. The non-severe form is characterized by high fever, lasting 2 to 5 days, likely accompanied by nausea, vomiting, rash, aches and pains, and/or leukopenia. The following warning signs may be present: abdominal pain, persistent vomiting, fluid retention, mucosal hemorrhage, malaise and drowsiness, hepatomegaly, high hematocrit, and thrombocytopenia. Beyond these symptoms and warning signs, severe dengue patients exhibit serious hemorrhage, organ impairment, and plasma leakage, which leads to fluid accumulation in the lungs and abdomen, causing respiratory distress and hypovolemic shock (World Health Organization, 2009). Nevertheless, the most common outcome of DENV infections, including in primary infections, is the asymptomatic infection, in which the virus and/or seroconversion can be detected in the complete absence of symptoms (Endy et al., 2011; Montoya et al., 2013; Grange et al., 2014; Duong et al., 2015). The DENV infection may also result in a subclinical/unapparent infection, with insufficient symptoms to be detected by existing surveillance systems and healthcare providers, although verifiable by viral and/or seroconversion detection methods (reviewed in Grange et al. (2014)). Using cartographic approaches, a total of 390 million worldwide DENV infections per year have been estimated, including almost 300 million that are clinically silent or mildly symptomatic (Bhatt et al., 2013).

## 5 The asymptomatic outcome

The reported proportion of asymptomatic DENV infections is highly variable, with different studies pointing to values that range from 15% to 98% of the infections (Burke et al., 1988; Potasman et al., 1999; Endy et al., 2002; Porter et al., 2005; Balmaseda et al., 2006; Mammen et al., 2008; Balmaseda et al., 2010; Baaten et al., 2011; Salje et al., 2018; Tsang et al., 2019; Velandia-Romero et al., 2020; De Santis et al., 2023) (summarized in **Table 1**), and as recently pointed out in a meta-analysis (Asish et al., 2023). These differences can be due to several viral and host factors, and combinations of both. As discussed below in more detail, different DENV serotypes can produce distinct proportions of asymptomatic cases, while the host’s genetic background may also affect the outcome of the infection, given its influence on cells’ permissiveness and immune response to the infection. Additionally, the immune status of the population, built over previous infections and vaccination plans, may also account for differences in asymptomatic proportions. To illustrate this, in regions with endemic dengue, secondary infections might result in a higher frequency of asymptomatic cases, since the protection window given by the presence of neutralizing antibodies lasts for approximately 2 years in heterologous infections (Montoya et al., 2013; Anderson et al., 2014), while in homologous infections it can be lifelong (Sabin, 1952). In this context, the time elapsed between major dengue outbreaks can also influence the infection outcome.

**Table 1:** Percentages of DENV infections that are asymptomatic, accessed by several studies.

Population	Study design	Year	Serotype	% asymptomatic	References	Observations
Thailand	Prospective cohort	1987	nd	87%	Burke et al., 1988	Pediatric
		1998-2000	nd	53%	Endy et al., 2002	Pediatric
		1998-2003	nd	65%	Salje et al., 2018	Pediatric
		2004-2005	nd	75%	Mammen et al., 2008	
Indonesia	Prospective cohort	2000-2002	nd	75%	Porter et al., 2005	
Israel	Travellers survey	-	nd	43%	Potasman et al., 1999	
Reunion Island	Prospective cohort	2019-2020	DENV-1, -2, -3	15%	De Santis et al., 2023	
Nicaragua	Prospective cohort	2001	DENV-2	93%	Balmaseda et al., 2006	Pediatric
		2002	DENV-1	85%		
Nicaragua	Prospective cohort	2004-2005	nd	95%	Balmaseda et al., 2010	Pediatric
		2005-2006	nd	83%		
		2006-2007	nd	94%		
		2007-2008	nd	75%		
Nicaragua	Estimated probability	2004-2010	DENV-1	90%	Tsang et al., 2019	Pediatric
			DENV-2	87%		
			DENV-3	76%		
			DENV-4	98%		
Colombia	Prospective cohort	2013-2015	nd	31%	Velandia-Romero et al., 2020	
China	Retrospective cohort	2013-2015	nd	97%	Liu et al., 2018	
Netherlands	Travellers survey	2006-2007	nd	64%	Baaten et al., 2011	

nd, not determined.

Box 1 **The host immune response**

Both innate and adaptive host immune responses are known to participate in the control of viral infections. In innate response, pattern recognition receptors such as membrane toll-like receptor 3 (TLR3) and TLR7, and cytoplasmic retinoic-acid inducible gene I (RIG-I)-like receptors (RLRs) are expressed by the phagocytic cells, representing one of the first lines of antiviral defense, through sensing viral nucleic acids [reviewed in Guo et al. (2018))] These sensor molecules trigger the activation of two important families of transcription factors - interferon regulatory factors (IRFs) and NF-kB - that prompt the production of type I interferons (IFN)α/β and inflammatory cytokines. A cellular antiviral status is therefore established in the infected and adjacent cells, that activates and recruits immune cells such as Natural Killer (NK), critical in the antiviral response (Shresta et al., 2004; Navarro-Sánchez et al., 2005; Bourne et al., 2007).

Regarding adaptative immunity, cellular immune response mediated by T-helper 1 (Th1) and cytotoxic T lymphocytes (CTLs) is the most effective antiviral mechanism once the virus enters the host cell. Induced by IL-12 secreted by DCs, Th1 lymphocytes produce inflammatory cytokines, namely IL-2, IFNγ, and TNFα, while CTLs produce IFNγ, contributing to the clearance of the infection. IL-12 also induces differentiation of CD8+ T cells into cytotoxic cells, triggering apoptosis of the infected cells. Humoral immunity, through neutralizing antibodies, is also thought to protect against DENV infections, by limiting infection dissemination and promoting viral clearance (Henchal et al., 1988; Clapham et al., 2015). Following a primary symptomatic DENV infection, DENV-specific IgM antibodies are detectable in serum 4 to 5 days after the onset of symptoms, remaining measurable for up to 3 months. Anti-DENV-IgG antibodies appear later, at about one week after the onset of symptoms, peaking several weeks after the infection, and then declining to lower levels that, nevertheless, remain detectable for decades (reviewed in Wahala and De Silva (2011); Salje et al., 2018). These antibodies are mainly from the IgG1 subclass (Koraka et al., 2001; Watanaveeradej et al., 2003; Hofmeister et al., 2011), indicating a Th1-based immune response. A large fraction of anti-DENV-IgG cross-react with all DENV serotypes and eventually with other flaviviruses. Effective protection is long-term against the homologous serotype from the primary DENV infection (Sabin, 1952), but only transient, up to 2 years, against heterologous serotypes (Montoya et al., 2013; Anderson et al., 2014). After that period, there is also a higher risk of developing severe dengue in heterologous infections, likely facilitated by the ADE mechanism [reviewed in Rothman (2011)].

Study design may also affect the estimated proportion of asymptomatic DENV infections. In line with the above-mentioned, when studying non-naïve populations, this proportion is expected to be higher, given the difficulty to differentiate between true asymptomatics in primary infections and the lack of symptoms in individuals within the partial immunity window, following previous DENV infection. In fact, while both situations are characterized by inapparent infections, the underlying mechanisms are different. An overestimation of asymptomatic frequencies may also result from unidentified symptomatic mild cases occurring without febrile illness, as most studies track symptomatic cases through surveillance of body temperatures (Burke et al., 1988; Endy et al., 2002; Balmaseda et al., 2006). Moreover, studies focusing on determining asymptomatic frequencies at a specific time point or short period are more likely to overestimate this proportion by failing to distinguish pre-symptomatic cases and true asymptomatic cases, contrarily to studies encompassing a longer follow-up of their participants (Ly et al., 2019). To minimize this effect, some studies only considered as asymptomatics those individuals who did not experience a documented febrile episode linked to DENV infection but had a 4-fold or greater increase in total DENV-specific antibody titers (Kuan et al., 2009; Balmaseda et al., 2010; Montoya et al., 2013). Additionally, some studies in hyperendemic regions with co-circulation of different flaviviral infections are also thought to have a higher proportion of asymptomatic DENV infections, likely due to induced cross-protective immunity (Ribeiro et al., 2018). Therefore, estimating the proportion of asymptomatic infections in previously naïve populations, and studying the mechanisms underlying the absence of symptoms in primary infections are, hence, wanted to a better understanding of DENV infection. These mechanisms may also shed light on the asymptomatic outcome of other flaviviral infections, given their several common features.

### 5.1 The importance of asymptomatic DENV infections

Asymptomatic infections are recognized as an important component of the overall burden of flaviviral infections. However, their realistic epidemiological weight is still largely unknown (Chastel, 2011). Given their generally high frequency, ignoring asymptomatic infections can result in an underestimation of the rate of infection and transmission in a community, inadequate evaluation of individual risk of severity in future infections, and improper implementation of control measures. Asymptomatics are less likely to disrupt their daily routines and, therefore, have a greater potential to contribute to the epidemic spread of the virus, and to its persistent circulation during interepidemic periods.

Humans with DENV asymptomatic infection were, for long, considered dead-end hosts, as it was assumed that they did not reach sufficiently high viremia to infect feeding mosquitoes. However, viremia of DENV asymptomatic infections is not easily established, since it is highly dependent on the day of the infection while, in symptomatics, the day of onset of symptoms is used as a reference. Nevertheless, Duong et al. (2015) were able to show that the average DENV viremia in asymptomatics is similar to that observed in symptomatics during the early and late viremic periods (2–3 days before or 5–8 days after symptoms onset, respectively). Moreover, these authors showed that pre-symptomatic or asymptomatic DENV-infected individuals are, at any given level of viremia, more infectious to mosquitoes than symptomatic individuals (Duong et al., 2015). Also, it was suggested that the strong humoral immune response and high cytokine levels developed during symptomatic infections likely reduce human infectiousness to mosquitoes during this period (Lambrechts et al., 2012). In fact, Nguyet et al. (2013) have previously associated the increasing number of days of illness and the rise of IgM and IgG titers with a reduced risk of human-to-mosquito DENV transmission. Moreover, a slower decay of viremia observed in asymptomatic infection (Matangkasombut et al., 2020) may lead to longstanding infectious reservoirs. The predictive models of Ten Bosch et al. (2018) estimated that only 1% of DENV transmissions are attributable to individuals presenting symptoms at the time of transmission. Subclinical DENV infections have, therefore, a significantly greater potential to contribute to viral transmission than previously recognized, including to the persistent circulation of DENV during interepidemic periods. For example, Jamjoom et al. (2016) hinted at the possible influence of asymptomatic infections on the establishment of dengue in Saudi Arabia where, until recently, reported clinical cases of dengue were sparse, but a high seroprevalence was detected. Moreover, an index-case study by Vazquez-Prokopec et al. (2023) suggested that transmission rates of DENV were higher in a scenario where inapparent infections are more frequent.

Asymptomatic infections can also play an important role in spreading the infection to new regions, where the arthropod vector is present in sufficiently high density to allow transmission. In the last few years, climate changes and the rise in temperature contributed to the increase of DENV infections. Climate changes stands as the major factor that could lead to a pandemic status of DENV, as it might allow for the vector to spread into areas outside its current niche. Predictive modelling, using prospective climate parameters, pinpoint an increased risk of DENV across the globe, including in Europe (Semanza and Shlomit Paz, 2021; Wang et al., 2023). Beyond the increase in the number of infected people and geographic range, the expansion to new areas will lead to new scenarios (genetics, lifestyles, environmental conditions) where status of the population in terms of asymptomatic or severe disease is basically unknown. Moreover, as asymptomatics are difficult to identify and control in terms of public health, transmission of DENV serotypes between borders is not only probable to increase, but also to seriously increase the number of heterologous infections.

When returning from dengue endemic regions, asymptomatic travelers are less likely to be detected than symptomatic and represent a possible entryway for the virus. For instance, the phylogenetic study of the DENV virus by Franco et al. (2015) indicated Venezuela as the most probable origin of the DENV1 strain responsible for the 2012 outbreak in Madeira, where the vector *Aedes aegypti* was present at considerable density. This origin was previously suggested by Wilder-Smith et al. (2014), considering the likelihood of introduction from dengue-endemic countries based on their dengue incidence and travel volume to Madeira. The large emigrant community from Madeira living in these countries, particularly Venezuela, frequently travels back to the island, so introduction via an asymptomatic traveler is a very likely scenario.

The repercussion of asymptomatic infections on viral transmission is not limited to the likelihood of infecting mosquitoes, since there are also well-documented episodes of passive human-human transmission through blood donations and transplants (Wilder-Smith and Schwartz, 2005; Chuang et al., 2008; Tambyah et al., 2008; Stramer et al., 2012). These are probably underestimated routes of transmission, where asymptomatic infections have an increased responsibility since infected individuals are less likely to donate blood while sick. Furthermore, the asymptomatic or misdiagnosed clinical illness in a patient following a transfusion or a transplant, the pre-existent homotypic or recent heterotypic immunity in the recipient, or an infection incorrectly attributed to mosquito transmission, are factors contributing to the sub-evaluation of human-human transmission (Petersen et al., 2013).

Lastly, we would like to highlight that asymptomatic infections are also thought to play a protective role against a symptomatic secondary infection. In a prospective pediatric cohort study, Montoya et al. (2013) showed that the time interval between an inapparent DENV infection and a subsequent inapparent infection was significantly shorter (2.2 years) than that for a secondary symptomatic infection (2.7 years), suggesting effective immune protection during a window period of almost 3 years, induced by the primary inapparent infection. Moreover, the mean time interval between two consecutive symptomatic infections was estimated at 3 years, thus suggesting that the window of cross-protection induced by inapparent, or symptomatic infections is similar (Montoya et al., 2013). Once the period of cross-protection is over, antibodies acquired during an asymptomatic infection might also contribute to a higher risk of severe forms of dengue during secondary heterologous infection. To our knowledge, it is not currently known whether a primary asymptomatic infection influences the severity of a subsequent heterotypic infection. The risk of developing severe dengue is linked to pre-existent anti-DENV antibodies (Morens and Halstead, 1987), being highest within a specific range of antibody titers (Katzelnick et al., 2017). As such, if the window of protection induced by a first asymptomatic infection is due to the presence of cross-reactive antibodies, then the likelihood of developing a severe symptomatic infection would be similar, regardless of whether the first infection was symptomatic or asymptomatic. However, antibody decay following an asymptomatic infection was shown to be faster than after a symptomatic infection (Luo et al., 2018). Therefore, the risk time window for severe dengue may be different following a primary asymptomatic infection. Although asymptomatic infections are largely unidentified, they are nevertheless coupled with an established antibody response, and a greater number of people beyond the identified symptomatics are at risk of developing severe forms of dengue following the protection window.

### 5.2 Factors influencing asymptomatic outcome

The asymptomatic DENV infections, as well as the severity of clinical manifestations in symptomatics, have been associated with viral factors, such as DENV serotype and load (Gubler et al., 1978; Vaughn et al., 2000; Balmaseda et al., 2006), and host factors, such as age, lipid profiles, genetic background (Lan and Hirayama, 2011; Xavier-Carvalho et al., 2013; Yeo et al., 2014), immune factors, including previous immunological experience (Gubler et al., 1978; Green et al., 1999; Vaughn et al., 2000; Navarro-Sánchez et al., 2005; Mathew and Rothman, 2008; Endy et al., 2011; Shresta, 2012; Tisoncik et al., 2012; Salje et al., 2018), and antibody-dependent enhancement (Morens and Halstead, 1987), as further detailed below. Also, factors associated with more severe outcomes, such as the co-circulation of other microorganisms or the existence of chronic diseases (Bravo et al., 1987; Kouri et al., 1989; Teixeira et al., 2015; Pang et al., 2017), might also be implicated in the outcome of the infection.

#### 5.2.1 Viral serotype and titer

Viral serotypes refer to closely related but genetically distinct viruses that, given their different surface antigens, trigger different responses in the human host. In the case of DENV, the four known circulating serotypes share approximately 65% of their genomes and, even within the same serotype, genetic variation may exist (Anoop et al., 2012). Despite these variations, the different serotypes result in the same disease and range of symptoms.

An association between DENV2 serotype and a higher proportion of severe dengue has been described (Balmaseda et al., 2006; Fried et al., 2010; Vicente et al., 2016). The efficient replication of this serotype, leading to high viral load is suggested as the main cause for more frequent severe manifestations (Vaughn et al., 2000; Thomas et al., 2008). In contrast, Yung et al. (2015) observed a significantly higher risk of severe dengue and higher viral load in DENV1 infections. On the other side of the spectrum lies the DENV4 serotype, which is typically associated with lower viral titters than other serotypes (Thomas et al., 2008) and milder forms of dengue fever (Nisalak et al., 2003; Thomas et al., 2014; Rocha et al., 2017).

Interestingly, Salje et al. (2018) observed a much higher proportion of asymptomatics in DENV4 infections compared to the other 3 circulating serotypes, a result equally supported by Tsang et al. (2019). These results suggest that the distribution and proportion of serotypes found in most symptomatic surveillance-based studies may not be representative of circulating DENV serotypes in a given region, since the infections with some DENV serotypes could be more frequently silent than others. It also suggests that the viral load, known to influence disease severity, might be partially responsible for asymptomatic infections, when at low levels. In fact, Duong et al. (2015) were able to estimate that DENV viremia in asymptomatics (4.75 ± 0.39 log_10_ cDNA copies/mL) is similar to that observed in the early and late viremic periods of symptomatic viremia, but significantly lower than during the viremic peak, between days 1 to 4 of illness (6.12 ± 0.17 log_10_ cDNA copies/mL). Also, a slower decay of viremia was observed in asymptomatic DENV infections when compared to symptomatic dengue, probably reflecting a slower rate of clearance (Matangkasombut et al., 2020).

Concurrent infection with multiple serotypes is also believed to affect dengue severity (Bharaj et al., 2008; Vinodkumar et al., 2013; Lardo et al., 2016; Soo et al., 2016), although controversy exists on this subject (Gubler et al., 1985; Loroño-Pino et al., 1999). To our knowledge, no studies thus far have reported the effect of co-infections with multiple DENV serotypes in the scenario of an asymptomatic outcome. Nevertheless, if co-infections influence the viral load, we could expect the outcome to be also affected. Other features usually not addressed are the size of the viral inoculum and the salivary contents introduced during the mosquito feeding, which may locally trigger different immune responses and lead to different adaptive immune response and/or viremias (Fain and Dobrovolny, 2020; Best et al., 2021; Ciupe et al., 2021).

In fact, the mosquito salivary gland contents introduced during the feeding process, in addition to promoting vasodilation and preventing clotting and platelet aggregation to facilitate blood intake (Manning and Cantaert, 2019), are also known to promote viral replication and to affect the host immune response (Schneider and Higgs, 2008; Pingen et al., 2017; Vogt et al., 2018). Components of the saliva may differ according to the mosquito species (Volf et al., 2000; Volf and Rohousová, 2001), geographical locations (Lanzaro et al., 1999; Volf et al., 2000; Ramalho-Ortigão et al., 2015), and whether the mosquito had previously fed on blood (Thangamani and Wikel, 2009; Bonizzoni et al., 2012). In addition, microbiome present in the mosquito saliva may be inoculated into the mammalian host, likely triggering innate receptors and influencing the immune response (Accoti et al., 2023). Therefore, small variations in the inoculum, in terms of saliva components and/or viral load, may contribute to differences in the outcome of infection.

Additionally, several studies have demonstrated that human exposition to mosquito bites leads to the development of antibodies against their salivary proteins, which are short-lived and, apparently, mosquito genus-specific (Orlandi-Pradines et al., 2007; Fontaine et al., 2011; Londono-Renteria et al., 2013). In endemic regions, the population is regularly bitten by mosquito vectors, particularly non-infected ones. Therefore, we can speculate that the immune response induced by pre-expositions to salivary proteins of non-infected mosquitos may influence the outcome of a subsequent DENV infection. Interestingly, Manning et al. (2022) found an increased risk of asymptomatic outcome to correlate with higher levels of anti-Ae. aegypti saliva antibodies, in a DENV-naive Cambodian children population. To clarify the possible role of pre-existing anti-mosquito saliva antibodies in the development of asymptomatic DENV infections, it would be interesting to compare their levels in symptomatic versus asymptomatic infected individuals.

#### 5.2.2 Age

Host age seems to be associated with the likelihood of asymptomatic or symptomatic outcome following a DENV infection, particularly in children. Thai et al. (2011) estimated, in a Vietnamese pediatric cohort, that the risk of developing symptoms increases with age, during both primary and secondary infections, being lower for children under 10 years old. Similar results were obtained by Tsang et al. (2019) in a prospective pediatric cohort in Nicaragua, with children over 8 years old being more than twice as likely to develop symptoms upon infection. Also, Montoya et al. (2013) found that the mean age at which symptomatic dengue occurs is higher than for asymptomatic DENV infection (8.4 vs. 7.2 years old, respectively), in Nicaraguan children. Thai children aged between 10 to 15 years old were also more prone to develop symptoms during primary infections than those aged 4 to 9 years old (Burke et al., 1988). In opposition, Endy et al. (2011) found no relationship between age and the ratio of asymptomatic: symptomatic infections in Thai children. This difference might be due to the limited number of studied children in the upper and lower age range, and inconsistencies in the study design.

Differences in the immune system between children and adults have been extensively described and may contribute to the higher prevalence of asymptomatics in younger children compared to older ones and adults. Variations in cytokine production (explained partially by epigenetic mechanisms; Bermick and Schaller, 2022), maturity of the cells of the adaptive system, and balance between effector and regulatory cells, have been identified between children and adults (reviewed in Chappell et al., 2021 and Semmes et al., 2021). The adjustments towards an adult’s immune system are progressive and, for most cellular components, occur up to around the age of 7 years (reviewed in Semmes et al., 2021). Interestingly, in specific anatomic regions, the innate immune system of children seems to respond differently and, in some ways, stronger than in adults (Loske et al., 2022; Yoshida et al., 2022). This may help to limit viral replication early on, resulting in mild or asymptomatic infections. For instance, a higher level of IFN on the steady state and a faster increase following SARS-CoV-2 infection were observed in the airways of children, who typically underwent asymptomatic or very mild infections (Loske et al., 2022; Pierangeli et al., 2022; Yoshida et al., 2022). This may be particularly relevant for high interferon-sensitive viruses that developed strategies to evade type I IFN-mediated antiviral activity. As a matter of fact, DENV was shown to be able to evade the immune response by blocking type I IFN (reviewed in Castillo Ramirez and Urcuqui-Inchima, 2015). If children’s skin-resident immune cells also display higher basal expression of genes associated with IFN signaling or other innate pathways, then we should expect an earlier *in situ* control of viral replication. This would lead to a lower dissemination of the virus and, consequently, to a lower inflammatory response. In addition, the more suppressed systemic adaptative response, typically observed in children, may also contribute to a higher resistance to disease, as discussed above. Trained immunity (Netea et al., 2020) can also have a contribution to the higher prevalence of asymptomatic infections in younger children, since repeated exposure to either viral infection or vaccination during childhood may lead to transient epigenetic changes in innate cells, leading to a faster IFN-response to DENV infection.

#### 5.2.3 Role of lipids

Lipid levels and their receptors may play a role in asymptomatic outcome as DENV infection and disease severity are believed to be directly linked to factors affecting lipid metabolism, serum lipoproteins, and their immunomodulatory effects. However, their role is not clear (Cui et al., 2013; Biswas et al., 2015; Durán et al., 2015; Voge et al., 2016; Marin-Palma et al., 2019). For instance, the presence of cholesterol and intact lipid rafts seem to be required for the activation of Jun NH(2)-terminal kinase and the p38 mitogen-activated protein kinases (MAPK) pathways during DENV infection of human macrophages (Ceballos-Olvera et al., 2010). Furthermore, DENV entry into mammalian cells is associated with the expression of receptors associated with lipid rafts (Reyes-Del Valle et al., 2005). In fact, DENV infection promotes significant changes in the cellular membranes of the host cells, providing structures for the replication complex (Junjhon et al., 2014) and, possibly, counteracting the host cellular innate immune response (Uchida et al., 2014). DENV is also known to promote the activation of autophagy in infected cells (Lee et al., 2008), which is critical for viral replication in several viral infections, since autophagosomes have been proposed to be sites of active viral RNA replication (Wileman, 2006; Deretic and Levine, 2009), and also because autophagy regulates lipid metabolism in infected cells. Heaton and Randall (2010) showed that DENV infection not only leads to the processing of cellular lipid droplets and triglycerides, but also that the depletion of lipid droplets correlates to an increase of autophagosomes and stimulation of β-oxidation in infected cells, providing energy for DENV virus replication. While some authors have shown this reduction of lipid droplets in response to DENV infection, acting as an “energy sink” that is tapped during viral replication (Heaton and Randall, 2010; Tongluan et al., 2017), others have reported an increase in lipid droplets (Samsa et al., 2009; Soto-Acosta et al., 2014), that act as binding sites for the DENV C protein during DENV assembly (Carvalho et al., 2012; Martins et al., 2012).

Evidence supporting the role of lipoproteins in the immunopathogenesis of dengue has been put forth by several studies that reported lower levels of high-density lipoprotein (HDL), low-density lipoprotein (LDL), and total cholesterol levels in more severe dengue cases (van Gorp et al., 2002; Suvarna and Rane, 2009; Biswas et al., 2015; Marin-Palma et al., 2019). In contrast, there is evidence of a direct association of serum apolipoprotein A-I (ApoA-I), the major protein component in HDL, with increased infectivity by Flaviviruses, as ApoA-I seems to facilitate their cell entry via scavenger receptor class B-type (SR-BI) (Li et al., 2013). For other members of the Flaviviridae family, there is also evidence that low-density lipoprotein receptors (LDL-R) may be the main entrance into cells (Agnello et al., 1999). Moreover, it was hypothesized that DENV may form lipoviroparticles, which would constitute a novel step in DENV life cycle (Faustino et al., 2014). For last, it is worth mentioning that, besides the well-known cytokine-induced changes in lipoprotein profile during infection (Grunfeld and Feingold, 1996), HDL also has immunomodulatory properties, through the regulation of inflammasomes and SR-BI expression in macrophages (Song et al., 2015; Thacker et al., 2016).

Evidence highlighting the interaction between lipid metabolism and protection against clinical dengue was reported by Sierra et al. (2017), whose genome-wide association study (GWAS) has identified two new genes – *OSBPL10* and *RXRA* - playing a role in infection resistance (more details in section *Host genetics*). Nevertheless, no comprehensive studies of lipid and metabolic profiles directed at asymptomatic individuals exist that could elucidate the role of lipids on the asymptomatic outcome.

Nutritional factors are known to have a significant impact on lipid profiles, as well as on the cellular components necessary for virus replication and the immunological health of the individual. Besides, some lipids have been demonstrated to have virucidal activity (Hilmarsson et al., 2007). Although studies on nutritional factors involving asymptomatic models or individuals are not available, nutritional status has been investigated as a way of predicting the severity of dengue infections (Ahmed et al., 2014; Te et al., 2022). However, the results of several of these studies have generated debate and some have been controversial. For instance, micronutrient supplementation appears to function as a supportive therapy that may lessen the probability of progressing from DENV infection to severe forms of the disease (Ahmed et al., 2014). Thus, nutrients may again play a role in asymptomatic dengue infection. Indeed, nutrient abnormalities have been described in asymptomatic human immunodeficiency virus (HIV-1) infection (Beach et al., 1992). Nonetheless, the scope of research in this area is still limited and wider population studies are needed, as they may help to understand the asymptomatic dengue infection and be potentially important in prevention and treatment.

Therefore, the full molecular mechanisms of DENV pathogenesis are far from being understood and no comprehensive studies of lipid and metabolic profiles directed at asymptomatic individuals, exist. Notwithstanding, given the evidence that lipid receptors seem to be the main entrance of flaviviruses and that low lipid blood levels showed to be associated with disease severity (Biswas et al., 2015; Leier et al., 2018; Alcalá et al., 2022; Alcalá and Ludert, 2023), we might speculate that asymptomatic individuals have lower lipid receptor expression levels and higher blood lipid concentrations than symptomatic. Studies involving asymptomatic individuals and models are needed to clarify the expected protective role of lipids and receptors.

#### 5.2.4 Host immune response

The study of the host immune factors underlying asymptomatic and subclinical arboviral infections is highly challenging, since many factors, such as differences in the host immune status and in the viral load between symptomatics and asymptomatics, can make inferences complex. In DENV infections, three main approaches have been used for this purpose, namely the comparison of gene expression profiles, cytokine serum levels, and *in vitro* stimulation of PBMCs between symptomatics and asymptomatics.

A few studies compared the gene expression profile of key molecules involved in the immune response of asymptomatic or symptomatic infections. Yeo et al. (2014) found a broad downregulation of host defense genes in asymptomatic infections when compared with symptomatic, and an up-regulation of a few specific genes. However, the patients analysed in this study were already in the convalescent phase, with undetectable viremia. The results are, therefore, more likely to correspond to feedback mechanisms aiming to restore homeostasis, with little direct contribution to the knowledge about host immune factors influencing the clinical outcome. On the other hand, Simon-Lorière et al. (2017) compared the gene expression profiles and serum levels of inflammatory cytokines in viremic asymptomatic and symptomatic children, considering their viral load. Although the asymptomatic group was quite small (n=9), no major differences were found between the two studied groups for genes involved in innate immune pathways such as antiviral immunity, activation of pattern recognition receptors, or IL-8 signalling. Accordingly, in Raghupathy et al. (1998), serum concentrations of the inflammatory cytokines IL-6, IL-8, IL-15, CCL3, and CCL4 were not different between viremic asymptomatic and clinical dengue patients, with the main observed differences being associated with dengue severity. In contrast, the pathways involving antigen presentation and activation of T and B cells were differentially expressed between the two groups studied by Simon-Lorière et al. (2017). In particular, these authors observed an up-regulation of genes involved in the antigen-presentation pathway, and in dendritic cell maturation in viremic asymptomatic children. Serum concentrations of IL-12 and IL-23, both indicative of antigen-presenting cells activation, were also increased, while the CD86 co-stimulatory molecule was significantly down-regulated in both CD14+ monocytes and Lin−CD11c+ dendritic cells. In addition, several T cell co-stimulatory pathways such as ICOS-ICOSL signaling in T helper cells, CD28/CTLA4 signaling in cytotoxic T lymphocytes, and expression of CD69 (an early activation marker of T cells), were up-regulated in asymptomatic children. In this same study, the serum concentration of IL-2, a cytokine associated with T cell activation and proliferation, was also increased, and the IL-2 signaling pathway was up-regulated in this group. Furthermore, immune response feedback mechanisms were also increased in the asymptomatic group (Simon-Lorière et al., 2017). In fact, higher activation of T cells has been previously associated with asymptomatic DENV infection when comparing *in vitro* stimulated PBMCs from healthy individuals, who subsequently developed either asymptomatic or symptomatic secondary DENV infections (Hatch et al., 2011; Friberg et al., 2018). Using this approach, the authors found a generally higher frequency of DENV-specific TNFα, IFNγ, and IL-2-producing T cells in the group that later developed secondary asymptomatic infections (Hatch et al., 2011), and a significantly lower secretion of IL-12, IL-2R, MIP-1α, RANTES, GM-CSF, and TNFα by PBMC from subjects who developed symptomatic infection (Friberg et al., 2018). In sum, asymptomatic DENV infections, at least secondary ones, seem to be associated with increased T cell activation and antiviral cytokine production coupled with proper immune response regulation.

Interestingly, a few studies addressing the symptomatic outcomes indicate that T cell activation may contribute to the pathogenesis of DENV infection through the production of inflammatory cytokines, leading to an exacerbated response (Chaturvedi et al., 2000; Mathew and Rothman, 2008; Rothman, 2011). However, other authors suggested that T cells, including CD8+ and CD4+ cytotoxic T cells, may play an important role in protection against severe dengue, allowing an efficient elimination of the virus without excessive immune activation and, consequently, without causing severe disease (Weiskopf and Sette, 2014; Weiskopf et al., 2015). It is possible that asymptomatic primary infections lie at the extreme of this response, with a higher anti-viral T cell activation coupled with proper control mechanisms, allowing viral clearance without leading to clinical symptoms. A limitation of the above-mentioned studies is that none was carried out in primarily infected populations and thus, the pre-existence of memory T cells, cross-immunity to different serotypes, and window of antibody clinical protection may have influenced the results. More recently, based on a study involving primary and secondary infections, Rouers et al. (2021) suggested that the outcome of a dengue symptomatic infection may result from an individual propensity, genetically and/or environmentally determined, to produce particular adaptative cell phenotypes. It is likely that the baseline adaptative cellular profile of an individual may also contribute to the asymptomatic outcome. This may be true not only for the adaptative immune cells phenotypes but also for the innate immune cells, as a consequence of the trained immunity.

Mouse models have been important for understanding the mechanisms of infection in many infectious diseases. Although it is difficult to establish a single model capable of reproducing every aspect of DENV natural infection in humans, wild-type, genetically engineered, and humanized mouse models have been shown to reproduce at least one or more features of the infection (reviewed in Chen and Diamond, 2020 and Coronel-Ruiz et al., 2020). Many of the existing models, such as AG129 (IFN α/β/γ R^-/-^) and A129 (IFN α/β R^-/-^) mice, were designed to overcome the type I IFNR signaling, which is known to be central for the natural resistance of mice to DENV infection (Yauch and Shresta, 2008). However, primary DENV infections in these models usually result in severe disease or even death (Shresta et al., 2005; Tang et al., 2011; Sarathy et al., 2015; Milligan et al., 2017). As for knockout mouse models lacking other specific molecules in the IFN cascades, despite being viremic, these do not show evident signs of disease and, thus, may allow to dissect the protective mechanisms against disease. For instance, using STAT1^-/-^ mice, Shresta et al. (2005) demonstrated that clearing of the initial viral load happens in a STAT1-dependent way. Still, the resolution of infection and protection against disease are based on STAT1-independent responses, and thus independent of the early control of viral replication. Other deficient mice such as Cardif^-/-^ (Perry et al., 2009) and STAT2^-/-^ (Perry et al., 2011), as well as wild-type mice transiently treated with MAR1-5A, an IFNAR1-blocking and non-cell-depleting antibody (Wilken et al., 2023), were shown to develop non-lethal viremia, together with no apparent signs of illness. Another interesting approach involved the conditional knockout of IFNAR expression in specific cell subsets of the mouse model. For instance, mice lacking IFNAR expression on either CD11c^+^ dendritic cells or LysM^+^ macrophages were susceptible to infection but also sufficiently immunocompetent to allow self-resolving viremia associated with a strong and fast CD8^+^ T cells response (Züst et al., 2014). Nevertheless, as far as we know, none of the above models have thus far objectively addressed the study of asymptomatic infections and therefore, deserve to be revisited or further manipulated in this context. Furthermore, in some studies, the interpretation of results regarding the asymptomatic outcome is made more difficult by the lack of a detailed description of disease signs or their absence, accompanied by the interchangeable use of the terms “protection against disease”, “protection against infection”, and the paucity of information regarding viremia in animals without symptoms.

Among many features of the disease pathogenesis, the above models also contributed to understanding the importance of the IFN signaling in resistance to infection. Therefore, it is possible that homologous genes in these pathways contribute to disease resistance in human populations, by limiting viremia at the early stage of infection. Other models, including humanized mice, may also be explored since they are likely better at mimicking human disease (Yauch and Shresta, 2008; Mota and Rico-Hesse, 2009), and thus may contribute to identifying and further elucidating the mechanisms of the adaptive response (Jaiswal et al., 2009; Jaiswal et al., 2012) possibly involved in the asymptomatic outcome.

It is noteworthy that using mouse models to study differences in the immune response between symptomatic and asymptomatic DENV infections requires the model to cover this full spectrum of infection outcomes. For this purpose, the recently described model using immunocompetent mice in which IFNAR1 is transiently blocked to allow infection (Wilken et al., 2023), appears to be promising. We can envisage that varying inoculum doses may result in different symptomatic versus asymptomatic outcomes, allowing to compare the underlying mechanisms of immune response.

#### 5.2.5 Host genetics

In the last decades, there has been increasing evidence that human genetic polymorphisms play a role in the activation of different immuno-pathological mechanisms involved in DENV infection and, therefore, in its different outcomes. However, most studies focused on identifying host genetic factors correlated to disease severity (Coffey et al., 2009; Lan and Hirayama, 2011; Xavier-Carvalho et al., 2017; reviewed in Cahill et al. (2018)), while the genetic determinants of asymptomatic DENV infection have been largely disregarded.

García et al. (2010) and Mohsin et al. (2015) accessed the contribution of the *FcϒRIIa-*H131R (rs1801274) polymorphism to the clinical manifestations of dengue in Cuban and Pakistani populations, respectively. In these studies, the 131H allele was found to increase the odds of developing clinical dengue, while 131R seems to confer protection against the clinical forms of the infection. Fc_ϒ_RIIa is an Fc receptor, which binds to the Fc component of the IgG antibodies, and this SNP is known to change the affinity of the Fc receptor to different IgG subclasses. Fc_ϒ_RIIa-131R receptors bind efficiently to IgG1 and IgG3 (Van Sorge et al., 2003), which are the predominant immunoglobulins during DENV infection (Koraka et al., 2001). Their interaction with opsonized DENV activates phagocytes, leading to a more efficient control of viral dissemination (Forthal and Moog, 2009). On the other hand, Fc_ϒ_RIIa-131H receptors seem to preferentially interact with IgG2 (Clark et al., 1991), thus favoring viral dissemination by ADE (Moi et al., 2010). Surprisingly, Noecker et al. (2014) suggested a protective role for Fc_ϒ_RIIa-131H in Mexicans, while associating Fc_ϒ_RIIa-131R with symptomatic dengue. According to the authors, the observed reverse association when compared to the findings of García et al. (2010) may be explained by differences between populations and study groups, either in other unstudied interacting genetic factors, age distribution, history of infection (data absent in García et al. (2010)), and/or viral serotype (DENV4 in Cuba and DENV1 in Mexico).

Several candidate genes of the type I IFN response pathway were analyzed by Silva et al. (2010) in Brazilian symptomatic DHF and DF and asymptomatic DENV infections. Two polymorphisms located at the 5’ end of *JAK1* gene were identified as the most significant. JAK1 is one of the first components of the type I IFN signaling pathway, known to control the response to flavivirus in mouse models (Perelygin et al., 2002). The associated SNPs may exert regulatory effects in *JAK1* expression and, thus, lead to the under-expression of type I IFN-induced genes, already described in severe dengue (Simmons et al., 2007). The work of Silva et al. (2010) focused mainly on the effect of genetic variants on disease severity, by comparing SNP frequencies of DHF with DF, but the authors claim that the association remains true, though weaker, when comparing DHF against asymptomatics (data not shown).

SNP haplotypes in *MBL2* have been significantly associated with dengue severity in Brazilian children with severe dengue when compared to asymptomatic DENV IgG-positive controls (Ornelas et al., 2019). This gene encodes for the mannose-binding lectin (MBL), a pattern-recognition receptor that acts in the first-line response to DENV, by triggering complement activation to promote viral neutralization (Avirutnan et al., 2011). *In silico* prediction has suggested that these SNPs act as expression regulators (Ornelas et al., 2019), likely resulting in the deficiency of MBL already observed in severe dengue (Alagarasu et al., 2012; Figueiredo et al., 2016).

Polymorphisms in the *IL4R* and *IL6R* cytokine receptors were also pinpointed as risk factors for clinical dengue in Colombian children (Useche et al., 2019), as these may introduce changes in the T cells signal transduction, and thus alter the activation of Th subtypes in dengue. The upregulation of IL4R has been previously described in asymptomatic DENV infection, when compared to clinical dengue (Yeo et al., 2014), suggesting its protective role. The *IL6R*-358Ala allele may result in lower expression of its soluble form sIL-6R on CD4+ T cells and monocytes (Ferreira et al., 2013), with consequent low activation of Th2 response and decreased recruitment of leukocytes, likely impairing an efficient antiviral response against DENV. Note, however, that Useche et al. (2019) based their conclusions on the comparison between DENV IgG-positive symptomatics and a control group lacking symptoms, but not tested by serology. As the study was conducted on a dengue-endemic region, both asymptomatic and non-infected individuals may have been included in the control group, certainly biasing the results.

The hypothesis-free GWAS by Sierra et al. (2017) highlighted polymorphisms in *OSBPL10* and *RXRA* as protective factors against DHF in Cubans with African ancestry, when compared with asymptomatics and population controls. Both genes are involved in the LXR/RXR activation pathway, which integrates lipid metabolism and immune functions, and are thus key players in viral entrance and replication, and in cytokine production. Their expression study in Cuban DHF cases and the analysis of a Thai dengue transcriptome dataset showed that both these genes are differentially expressed along disease progression. For the top-associated *OSBPL10* SNPs, the authors have then determined the most common haplotypic combination in European and African (mostly sub-Saharan) reference populations from 1000 Genomes (1000 Genomes Project Consortium et al., 2012), and analyzed their expression based on the available datasets from the 1000 Genomes transcriptome (Lappalainen et al., 2013). In brief, the expression data suggests that the OSBLP10 haplotype affects its mRNA expression, with significantly lower levels (by half) in Nigerian Yoruba as compared to European and European-ancestry reference populations. Lastly, an *in vitro* knockdown of OSBPL10 assay, followed by DENV2 infection, revealed a significant reduction of viral replication, thus providing functional proof that the low expression of this gene likely contributes to the natural resistance against clinical dengue. While the results cannot be extended to all African populations, an approximation can be made to the genetic profile of Central and Western African populations and African-American descendants from transatlantic slavery.

The results of some of the above-mentioned studies remain discrepant, while others lack functional evidence for their positive associations. Differences in the studies’ design and data analyses exist, many times without clear biological reasoning. For instance, the influence of genetic polymorphisms in dengue severity is often assessed by comparing their frequencies in the severe forms DHF/DSS against those in asymptomatic DENV infections, instead of comparing symptomatic groups with different outcomes. In our understanding, the genetic mechanisms influencing dengue severity may be distinct from those underlying the clinical protection in asymptomatic DENV infection. Moreover, we recognize another important limitation: most studies were undertaken in dengue-endemic regions and, as retrospective studies, were unable to distinguish between primary and post-primary infections. This will surely hinder the study of natural, genetically-conferred clinical protection in primary infections.

## 6 Concluding remarks

As for many flaviviral infections, DENV asymptomatic infections represent the unseen part of the iceberg. Likewise, arthropod-borne viral infections caused by non-flaviviruses can also result in asymptomatic outcome, even on those in which humans act as dead-end hosts. For instance, infection by Crimean-Congo Hemorrhagic Fever or the Rift-Valley Fever viruses, both Bunyaviruses, and Chykungunya, Western-, Eastern- and Venezuelan Equine Encephalitis, or Sindbis viruses, all of them alphaviruses, are known to result in an asymptomatic outcome in 4 to 96% of infected humans (Laine et al., 2004; Bodur et al., 2012; Thiberville et al., 2013; Phelps et al., 2017; Wright et al., 2019). It is, therefore, crucial to understand their real impact on the spread of the pathogenic agent and its persistent circulation during interepidemic periods, as well as their contribution to herd immunity. However, asymptomatics are unlikely to play a major role in viral transmission on infections in which humans act as dead-end hosts. Nonetheless, all human arthropod-borne viral infections resulting in asymptomatic outcome may share common features with asymptomatic DENV infections. It is equally imperative to determine the individual immune status induced by asymptomatic DENV infections, the risk of long-term sequalae, and if secondary heterologous infections pose an increased susceptibility of asymptomatics to more severe forms of the disease, as widely described for symptomatics. Although not an arbovirus, the very recent example of the SARS-CoV-2 illustrates how challenging it can be to understand these aspects. In fact, the perception of the impact of asymptomatic infections in viral transmission and individual health changed across the pandemic.

**Figure 2:**
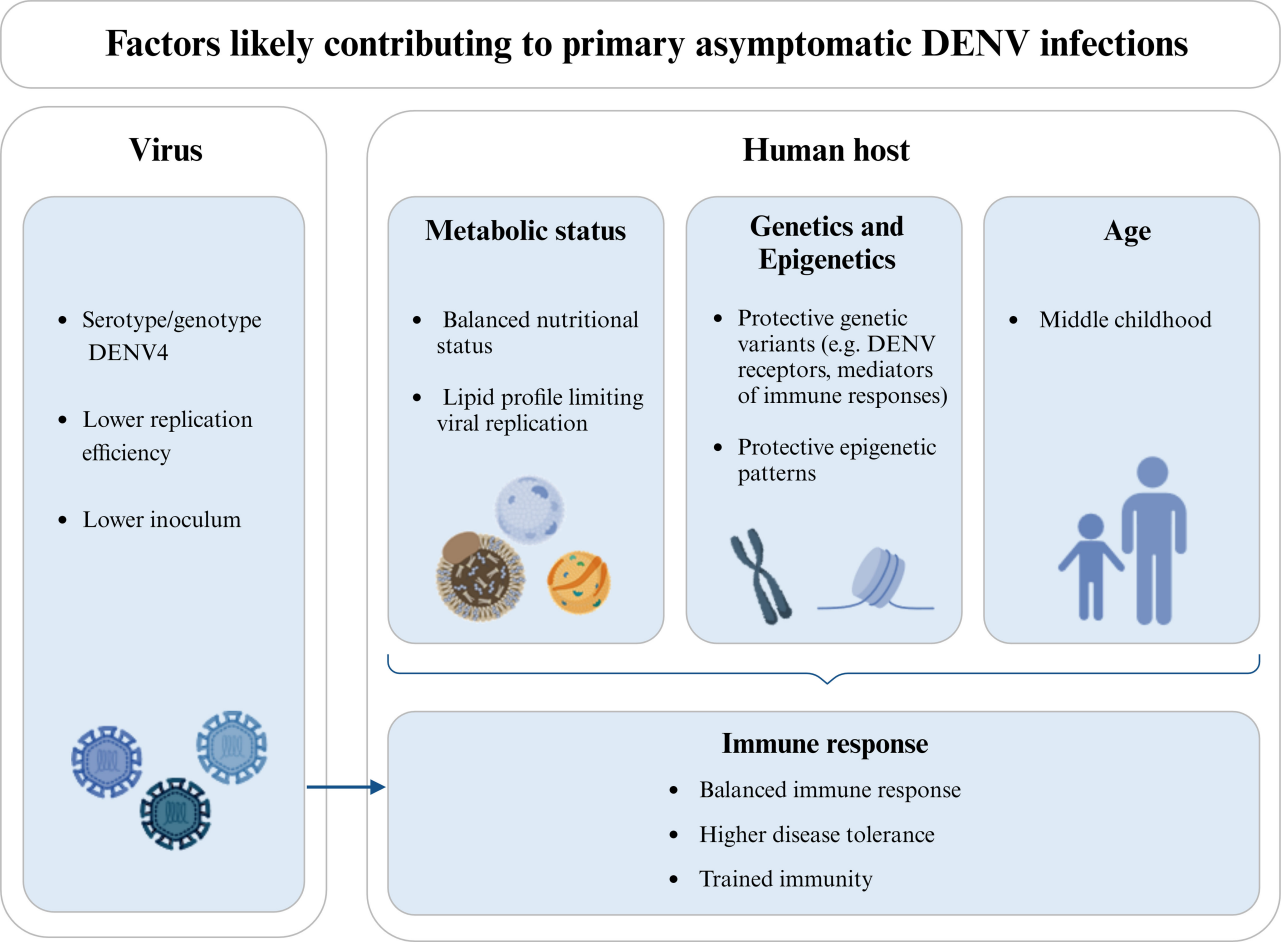
Factors likely influencing the clinical protection in individuals experiencing a DENV primary infection. Different viral serotypes/genotypes may have distinct abilities to infect human cells and/or replicate, thus influencing the viral load and the course of infection. DENV4 is more commonly referred to as causing a higher proportion of asymptomatic infections and is also associated with a lower replication efficiency. In the same line, the size of the viral inoculum introduced during the mosquito blood feeding may locally trigger different immune responses and/or lead to different viremias. Additionally, the metabolic status of the host, and in particular his nutritional status and lipid profile may contribute to the asymptomatic outcome in a primary DENV infection, either by regulating viremia or modulating the host’s immune response and/or epigenetic patterns. Host genetic variants and differential expression levels (likely mediated by epigenetic patterns) have also been associated with protection against clinical dengue. This is the case of genes coding for receptors used by DENV to infect human cells and mediators of the immune response or disease tolerance. In turn, age influences the host’s metabolic status, epigenetic patterns, and immune response, being commonly found in the literature that asymptomatic DENV infections are more frequent in children up to middle childhood, when compared to upper childhood and adulthood. The host’s immune response, including mechanisms of disease tolerance and trained immunity, as an epigenetic reprogramming of innate immune cells induced by previous exposition to unrelated pathogens, can also influence the outcome of DENV infection. Also, the protective immune mechanisms primed by the pre-exposition of the host to proteins of the mosquito saliva should be here considered. Note that the host’s metabolic status, genetics, epigenetics, and age are not independent factors and, together with viral factors, can as well influence the immune status of the host. Diagram was made using BioRender.com.

For a clearer picture, it is also essential to study the biological mechanisms likely conferring protection against clinical manifestations (resumed in **Figure 2**), observed in many flaviviral diseases. Why do some people become ill with a primary infection, while others are tolerant to the disease and capable of clearing the virus without changing their health status? Most studies concentrated their efforts on the determinants of severe versus mild infections, while asymptomatic infections remain disregarded. Furthermore, and especially in endemic areas, immunity acquired through a previous DENV infection can lead to clinical protection in a subsequent infection. This factor hinders the identification of biologically-defined asymptomatics and must be accounted for in the study design, since the mechanisms leading to the absence of symptoms within a window of immune protection are surely different from those underlying asymptomatic primary infections.

Additional evidence from adequately designed genetic and functional studies that objectively address asymptomatic infections is demanded, preferably in populations with unique epidemiological situations such as a primary infection by a single DENV serotype. These may strongly contribute to a better understanding of the mechanisms of disease, by evidencing genes, cellular pathways, and lipid profiles contributing to the natural protection against dengue. Hence, comprehensive knowledge of the physiological and immunological processes balancing the control of the flaviviral agent and the health status of the human host, in asymptomatic infections, can be crucial for the rational design of effective therapies, and are likely common to other infectious diseases.

## Author contributions

PH: Writing – original draft, Writing – review & editing. AR: Funding acquisition, Writing – original draft, Writing – review & editing. HC: Funding acquisition, Writing – original draft, Writing – review & editing. PS: Writing – review & editing. AV: Conceptualization, Funding acquisition, Writing – original draft, Writing – review & editing.
